# Effects of acute alcohol administration on endocannabinoids and relation to subjective effects

**DOI:** 10.1007/s00213-025-06843-6

**Published:** 2025-07-25

**Authors:** Gavin N. Petrie, Raegan Mazurka, Elisabeth R. Paul, Niclas Stensson, Bijar Ghafouri, Matthew N. Hill, Markus Heilig, Leah M. Mayo

**Affiliations:** 1 Department of Psychiatry, University of Calgary, Calgary, AB T2N 1N4 Canada; 2 Hotchkiss Brain Institute, University of Calgary, Calgary, AB Canada; 3 Mathison Centre for Mental Health Research and Education, University of Calgary, Calgary, AB Canada; 4 Center for Social and Affective Neuroscience, Department of Biomedical and Clinical Sciences, Linköping University, Linköping, Sweden; 5 Center for Medical Image Science and Visualization (CMIV), Linköping, Sweden; 6 Pain and Rehabilitation Centre, Department of Health, Medicine and Caring Sciences, Linköping University, Linköping, Sweden; 7 Department of Psychiatry, Linköping University Hospital, Linköping, Sweden

**Keywords:** Endocannabinoids, Alcohol, Subjective effects, 2-AG

## Abstract

**Rationale:**

Harmful alcohol use remains a significant global public health challenge. Examining variability in the acute subjective effects of alcohol and related neurobiological mechanisms may advance the understanding of susceptibility to harmful alcohol use. Research suggests the endocannabinoid (eCB) system may play an important role in mediating the reinforcing effects of alcohol. This study examined the relationship between alcohol-induced changes in eCB concentrations and the subjective psychoactive effects of acute alcohol consumption.

**Method:**

Healthy social drinkers (n = 28, aged 20–35 years) participated in a within-subjects, single-blind, placebo-controlled laboratory alcohol challenge study. Alcohol (0.6 g/kg; with 20% adjustment for women) and placebo sessions were counterbalanced. Subjective alcohol effects were assessed from self-report questionnaires administered pre- and post-dosing, including the Biphasic Alcohol Effects Scale (BAES), Drug Effects Questionnaire (DEQ), and Profile of Mood States (POMS). The eCBs, N-arachidonoylethanolamine (anandamide; AEA) and 2-arachidonylglycerol (2-AG), were assessed from blood plasma taken throughout the dosing session.

**Results:**

Acute alcohol was associated with an overall decrease in 2-AG concentrations compared to placebo. Further, we found that a *drop* in 2-AG concentrations was associated with less drug ‘liking’ and feelings of ‘friendliness’, whereas under placebo conditions, a *rise* in 2-AG was associated with a smaller decrease in feelings of ‘stimulation’ (e.g., feeling energized, talkative). Alcohol did not significantly affect AEA concentrations.

**Conclusion:**

Our study provides the first evidence that eCBs may contribute to individual differences in sensitivity to alcohol's reward-related mechanisms by influencing subjective experience, offering insight into the potential role of eCBs in the processes underlying harmful alcohol use.

**Supplementary Information:**

The online version contains supplementary material available at 10.1007/s00213-025-06843-6.

## Introduction

Alcohol is one of the oldest and most widely consumed psychoactive substances in human history. While often consumed without consequence, harmful alcohol use remains a major global public health challenge (World Health Organization 2018). Excessive alcohol use can lead to both immediate harms, including injuries from accidents, aggression, and violence, as well as long-term consequences, such as alcohol use disorders (AUDs) and increased risk of serious medical conditions like liver disease. A key to understanding susceptibility to harmful alcohol use lies in improving understanding of the acute subjective effects of alcohol (Morean and Corbin 2010) and the neurobiological mechanisms that underlie these effects. The endocannabinoid (eCB) system has emerged as a potential modulator of the rewarding aspects of alcohol consumption, which may help to explain individual differences in alcohol use and outcomes.

The subjective response to alcohol varies across individuals, and even within a single drinking episode. In humans, the subjective psychoactive effects of alcohol are biphasic, with stimulant effects typically occurring during the rising limb of the blood alcohol concentration curve, while sedative effects emerge during the descending limb (Martin et al. 1993; Pohorecky 1977). These biphasic effects likely contribute to alcohol’s rewarding properties. The stimulant phase may be linked to hedonic reinforcement, where the “liking” effects increase motivation to seek alcohol. In contrast, the sedative phase may promote negative reinforcement, through alleviation of stress, anxiety, or other aversive states (Boyd et al. 2017; Gilpin 2008). At a neurobiological level, alcohol's central nervous system (CNS) depressant actions are related to its sedative and anxiolytic effects, and are primarily mediated through direct activity at glutamatergic and GABAergic receptors. In contrast, the psychostimulant-like effects are driven predominantly by downstream dopaminergic and opioid systems (see MacKillop et al. 2022). The eCB system, which modulates synaptic transmission through retrograde signaling, may interact with these pathways to influence alcohol’s subjective effects by regulating the release of key neurotransmitters involved in its stimulant and sedative properties (Wolfe et al. 2022).

The eCB system includes two main endogenous ligands – N-arachidonoylethanolamine (anandamide; AEA) and 2-arachidonoylglycerol (2-AG)—which bind primarily to cannabinoid receptor 1 (CB1) and the more peripherally located cannabinoid receptor 2 (CB2). CB1 is the brain’s most abundant GPCR, expressed widely in stress- and reward-related regions (Devane et al. 1992; Morena et al. 2016; Pacher et al. 2006). AEA and 2-AG are synthesized on demand in response to neuronal activity. AEA is synthesized in post-synaptic neurons by N-acyl phophatidylethanolamine phospholipase D (NAPE-PLD; Cadas et al. 1997) and degraded by fatty acid amide hydrolase (FAAH; Cravatt et al. 2001). 2-AG is synthesized via diacylglycerol lipase (DAGL; Sugiura et al. 1995) and degraded pre-synaptically by monoacylglycerol lipase (MAGL; Dinh et al. 2002).

Preclinical studies provide robust evidence for the eCB system playing a role in regulating alcohol consumption, specifically through the CB1 receptor’s interaction with the mesolimbic dopamine pathway, a critical circuit mediating alcohol’s rewarding effects (Charlet et al. 2013; Sagheddu et al. 2015) (see Kunos 2020 for full review). For example, studies show that CB1 receptor agonists, such as WIN 55212–2, increase alcohol intake (e.g., Colombo et al. 2002; Linsenbardt and Boehm 2009), while CB1 receptor antagonists suppress alcohol consumption (e.g., Economidou et al. 2006). In addition, elevating eCB signaling by inhibiting eCB degradation, such as through pharmacological inhibition or genetic deletion of FAAH, has been shown to increase alcohol preference (e.g., Basavarajappa et al. 2006; Blednov et al. 2007), further supporting the role of the eCB system in promoting alcohol’s rewarding properties. Notably, CB1 receptor knockout mice exhibit markedly reduced alcohol consumption and fail to show alcohol-induced dopamine release in the nucleus accumbens, highlighting the connection between CB1 receptors and dopaminergic mechanisms in mediating alcohol's rewarding effects (Hungund et al. 2003).

Evidence also implicates the eCB system in alcohol’s sedative effects. FAAH knockout mice, which show enhanced AEA signaling, exhibit increased alcohol preference alongside reduced sensitivity to alcohol’s sedative and motor-disruptive effects (Blednov et al. 2007; Vinod et al. 2008). In contrast, CB1 receptor knockout mice show the opposite pattern: reduced alcohol preference and heightened sensitivity to alcohol’s sedative effects (Naassila et al. 2004). Taken together, animal studies suggested that the eCB system may play a role in modulating alcohol’s biphasic and reinforcing effects.

However, human findings are less clear. Consistent with animal models, genetic variation in FAAH activity has been associated with drinking behavior. The FAAH C385A (rs324420) variant A allele, which reduces FAAH activity and increases AEA, is associated with greater and more hazardous alcohol consumption, more severe alcohol dependence (Best et al. 2021; Sloan et al. 2018), and greater likelihood of concurrent drug and alcohol use (Sipe et al. 2002). However, blockade of CB1 receptors using the inverse agonist rimonabant did not affect alcohol use in people, neither under laboratory conditions, nor in clinical trials (George et al. 2010; Soyka et al. 2008). While exogenous activation of CB1 receptors does not appear to influence alcohol consumption in humans, growing evidence suggests that chronic alcohol consumption does impact availability of AEA and 2-AG (Elliott et al. 2025). Thus, while animal findings and some human research implicate the eCB system in harmful patterns of alcohol use, research is needed to examine how endogenous cannabinoid signaling relates to the subjective psychoactive effects of acute alcohol in humans, which are theorized to contribute to its reinforcing properties (King et al. 2011).

The goal of the current study was to investigate the relationship between alcohol-induced changes in eCB concentrations and the subjective psychoactive effects of acute alcohol administration in healthy social drinkers. Understanding this relationship is critical to uncovering how eCBs might contribute to risk or protection against harmful drinking (King et al. 2021, 2011).

We hypothesized that changes in eCB concentrations would be associated with alcohol’s stimulant, sedative, and reinforcing subjective effects (i.e., liking and wanting). Given the putative role of the eCB system in affect regulation (Gowatch et al. 2024; Lutz 2009), we also explored the relationship of eCB changes with a broad range of subjective feeling states. Due to limited and mixed evidence on whether alcohol increases or decreases eCB levels (humans: Feuerecker et al. 2012; Joosten et al. 2010; Schrieks et al. 2015; Sloan et al. 2022); (animals: Ceccarini et al. 2013; Ferrer et al. 2007; McPartland et al. 2014; Pavón et al. 2019), we refrained from forming directional hypotheses for these associations. However, a secondary objective of this exploratory study was to further clarify the directionality of alcohol-related changes in eCBs.

## Materials and methods

### Participants

Participants were healthy adults, aged 20 to 35 years (Table 1), recruited through flyers and social media. Exclusion criteria included a lifetime diagnosis of alcohol dependence, recent (within the past month) use of illegal substances or psychiatric medication, current DSM-IV Axis I disorders other than substance use, and lifetime diagnosis of psychotic or bipolar disorders determined by the Mini International Neuropsychiatric Interview (MINI). Participants were also excluded if they had any contraindications for MRI scanning (neuroimaging results to be published in a future report). The study was approved by the Regional Ethics Review Board in Linköping, Sweden (Dnr 2016/497–31). All participants provided written informed consent.

**Table 1:** Participant demographics (*n* = 28)

Age, *Median* (IQR)	23.5 (21–27.25)
Sex (female), *n*(%)	13 (46.43)
Marital Status (married), *n*(%)	3 (10.71)
Education (Bachelor’s degree or higher), *n*(%)	8 (28.57)
Employment (full-time), *n*(%)	12 (42.86)
AUDIT score, *Median* (IQR)	6 (3.5–7.5)
Average drinks per drinking day, *Median* (IQR)*	6 (3–6)
Total drinking days, *Median* (IQR)*	4 (2.5–6)
Binge drinking days, *Median* (IQR)*	1 (0–2)

*Data from Timeline Followback interview was missing for *n* = 1. Marital status was missing for *n* = 2. *IQR* Interquartile range

Of the 32 participants who attended the laboratory sessions, 28 participants had complete blood sampling and self-report measures and were included in the current report (*n* = 4 missing blood samples due to loss of intravenous catheter patency). Due to optimization of our extraction method for AEA, 2-AG could not be detected in a sufficient number of samples for two participants, leaving a sample of *n* = 26 for these analyses. An additional participant was missing 2-AG only for T + 150 min in the Placebo condition and was retained in analyses, where possible.

### Experimental Procedures

The study included a screening session and two 4–5 h experimental sessions. At screening, demographic and alcohol drinking behavior were collected using a Timeline Followback interview covering the past month (Sobell and Sobell 1992) and the Alcohol Use Disorders Identification Test (AUDIT; Saunders et al. 1993).

**Fig. 1:**
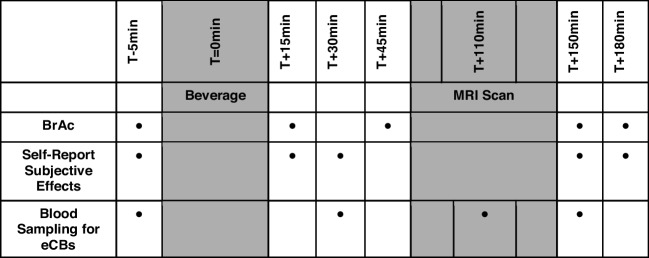
Timeline of procedures and data collection for Alcohol and Placebo experimental sessions. BrAC = Breath alcohol concentrations. eCBs = Endocannabinoids

Experimental sessions followed an identical procedure (see Fig. 1) that included an MRI scan (neuroimaging data not reported here). Participants were randomized to receive alcohol or placebo at session 1, and the alternate at session 2 (1–7 days following session 1). Upon arrival, an intravenous catheter was placed for repeated blood sampling and participants consumed a beverage (alcohol or placebo). The alcoholic beverage contained cranberry juice mixed with vodka (0.6 g/kg alcohol for males, with a 20% reduction for females to account for body water content differences Frezza et al. 1990; Sutker et al. 1983). The placebo contained cranberry juice with a 1% alcohol float to mask the smell and taste. To reduce potential expectancies, participants were informed that beverages might contain a placebo, alcohol, caffeine, or a sedative. Breath alcohol concentrations (BrAC), blood samples, and self-report questionnaires were collected before beverage intake and repeatedly throughout each session to assess intoxication levels, eCB concentrations, and subjective effects.

### Subjective feeling states and effects of alcohol

Participants completed the Biphasic Alcohol Effects Scale (BAES; Martin et al. 1993), the Drug Effects Questionnaire (DEQ; Morean et al. 2013), and the Profile of Mood States (POMS; (McNair et al. 1992) to assess self-reported changes in subjective effects including stimulant, sedative, and reinforcing effects of alcohol (see Fig. 1 for timing of questionnaire administration). The BAES measures the biphasic effects of alcohol, capturing both stimulation (BAES-STIM; e.g., feeling energized, talkative) and sedation (BAES-SED; e.g., feeling sluggish, heavy-headed) responses. A modified version was used to avoid disclosing the beverage contents (Rueger et al. 2009). The DEQ consists of 100 mm visual analog scales assessing feeling the effects of alcohol (DEQ-Feel), feeling high (DEQ-High), liking the effects (DEQ-Like), and wanting more (DEQ-More). The POMS assesses transient emotional states across multiple dimensions including Anxiety, Anger, Vigor, Fatigue, Depression, Confusion, Elation, and Friendliness.

We examined subjective effects of Alcohol/Placebo administration both pre- and post- T + 110 min to coincide with the change in eCBs observed at T + 110 min (see Results). Pre-T + 110 min change in subjective effects was calculated as: [Mean of (T + 15 min) and (T + 30 min)] – T-5 min. Post-T + 110 min change in subjective effects was calculated as: (T + 150 min) – (T-5 min). Positive values indicate an increase in subjective feeling state relative to baseline (e.g., more ‘liking’), whereas negative values indicate a decrease in the subjective feeling state (e.g., less ‘liking’). Values closer to 0 represent less absolute change.

Participants also completed the State-Trait Anxiety Inventory – State Scale (STAI-S; Spielberger et al. 2017) to assess state anxiety levels at the beginning of each study visit (scores range from 20–80; higher scores indicate greater anxiety).

### Endocannabinoids and blood alcohol

BrAC (mg/L) was measured via breathalyzer. Peripheral concentrations of the two primary eCBs – AEA and 2-AG – were measured from blood plasma collected via an intravenous catheter (see Fig. 1 for sampling timepoints). ECBs were quantified using liquid chromatography tandem mass spectrometry (LC–MS/MS), as previously published (Stensson et al. 2018). Analysis of the two eCB-like N-acylethanolamides (NAEs) – oleoylethanolamide (OEA) and palmitoylethanolamide (PEA) – are reported in the Supplemental Material.

In addition to comparing absolute eCB concentrations, to capture the dynamic response of the eCB system following alcohol, we calculated proportional change in eCBs relative to individual baseline: [(Timepoint) – (T-5 min)]/(T-5 min). Positive values reflect a rise in eCBs between timepoints, whereas negative values reflect a drop in eCBs. Larger values indicate greater change, whereas values near zero indicate lesser rise/drop.

### Statistical analysis

We used STATA 17 for our statistical analyses and R for data visualization.

To measure the effects of alcohol administration on BrAC and subjective intoxication over time in the Alcohol versus Placebo conditions, we used Wilcoxon matched-pairs signed-ranks tests. These tests were also used to assess alcohol-related changes in eCBs by comparing concentrations in the Alcohol versus Placebo condition at each timepoint. Additionally, to test whether the proportional change in eCBs differed from 0—and thereby determine whether eCB levels increased or decreased overall within the Alcohol and Placebo conditions—we used one-sided Wilcoxon signed-rank tests. Comparisons between the Alcohol and Placebo conditions alone only indicate whether eCB levels differ between conditions, without revealing the direction of change relative to baseline within each condition.

We used Spearman’s rank correlations to assess associations between proportional change in eCB concentrations and subjective responses to Alcohol.^1^ The Placebo condition served as a control to ensure that correlations of eCBs and subjective responses are specific to the effects of Alcohol and not an artifact of time or the study procedures.

## Results

### Participant characteristics and baseline

Participant characteristics for the sample (*n* = 28) are summarized in Table 1. Median scores on the AUDIT indicated most participants were in the “Low Risk” category (i.e., AUDIT ≤ 7). The scores from seven participants (25%) fell outside this range.

Median STAI-S scores at baseline were consistent with low levels of anxiety for both the Alcohol and Placebo sessions (*Median* = 33, *IQR* = 30–37, range 24–47 and *Median* = 33, *IQR* = 28.5–38, range 22–42).

### Change in BrAC and subjective intoxication over time for alcohol versus placebo conditions

**Fig. 2:**
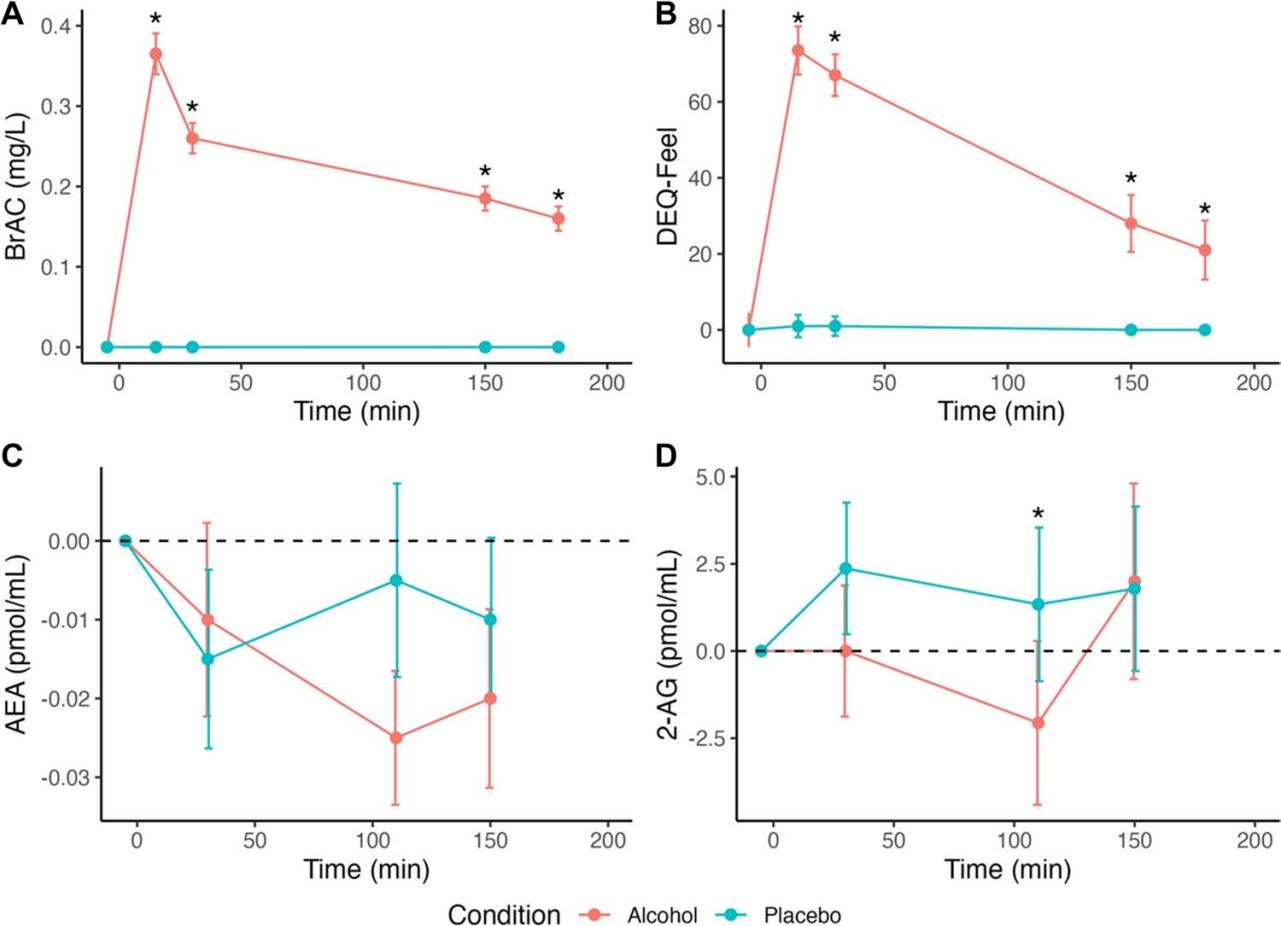
Breath alcohol concentration (BrAC) and self-report of “feeling” the effects of alcohol (DEQ-Feel) both peaked 15 min after beverage consumption and were greater in the Alcohol versus Placebo condition at all post-beverage time points (**A** and **B**). Absolute concentrations of 2-AG were lower in the Alcohol condition compared to Placebo at 110 min after beverage consumption (**D**). No significant differences emerged for AEA (**C**). Plots A and B depict median BrAC and DEQ-Feel, respectively. Plots C and D depict median absolute change in AEA and 2-AG concentrations relative to baseline levels, respectively. The dashed line represents baseline. Error bars denote standard error of the median. *p <.05 Wilcoxon Signed-Rank Test

BrAC concentrations confirmed the expected timing of the ascending and descending limbs for the Alcohol condition, such that BrAC peaked at T + 15 min and were consistent with the dose administered (Fig. 2; *Median* = 0.37, *IQR* = 0.43-0.31, Range = 0.23-0.66 mg/L). Further, self-report on “feeling” the effects of alcohol (DEQ-Feel) confirmed that subjective intoxication was achieved with the moderate-to-high alcohol dose. Both BrAC and DEQ-Feel were statistically significantly greater in the Alcohol versus Placebo condition at all post-beverage time points (BrAC *Z*s > 4.54, *p*s < 0.001 and DEQ-Feel *Z*s > 4.20, *p*s < 0.001; Fig. 2).

Consistent with the biphasic effects of alcohol, the stimulatory effects of alcohol peaked at T + 30 min and the sedative effects at T + 150 min. (see Supplemental Materials).

### Change in endocannabinoids over time for alcohol versus placebo conditions

**Fig. 3:**
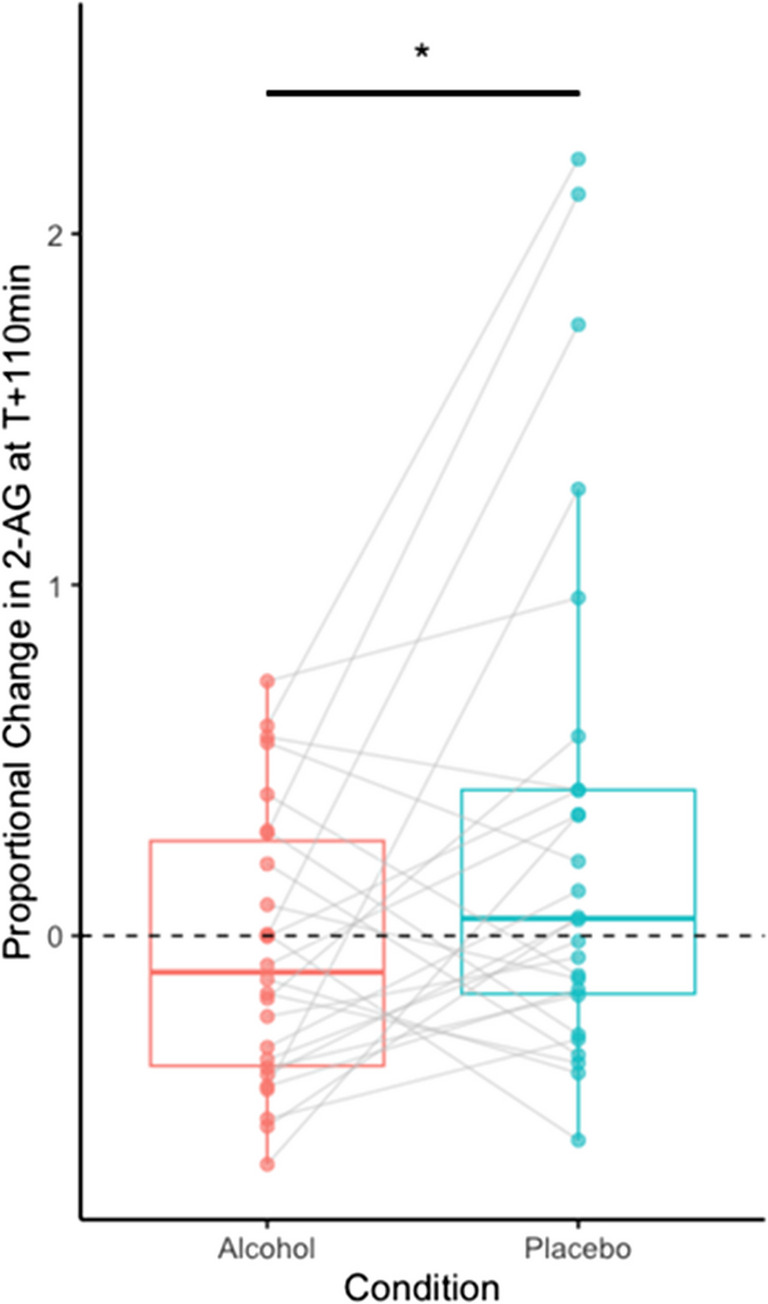
Proportional change in 2-AG was lower in the Alcohol condition compared to Placebo at 110 min after beverage consumption. Boxplot depicts median and interquartile range of proportional change in 2-AG concentrations compared to baseline. Lines connect individual participants across Alcohol and Placebo conditions. Dash line at 0 represents no change from baseline. *p <.05 Wilcoxon Signed-Rank Test

*2-AG.* Alcohol intake reduced 2-AG concentrations, as indicated by lower 2-AG in the Alcohol condition compared to Placebo at T + 110 min, *Z* = −2.12, *p* = 0.034 (Fig. 2). The effect of alcohol on 2-AG concentrations was also reflected in greater proportional change in 2-AG compared to baseline in the Alcohol condition compared to Placebo at T + 110 min, *Z* = −1.97, *p* = 0.049 (Fig. 3). There were no absolute or proportional differences between Alcohol and Placebo conditions at baseline, T + 30 min, or T + 150 min, *p*s > 0.200.

To evaluate whether 2-AG levels changed overall within each condition, we tested whether the proportional change at T + 110 min differed from 0. These analyses showed no significant difference from 0 in either Alcohol or Placebo conditions, *Z* = −0.58, *p* = 0.559 and *Z* = 1.33, *p* = 0.182, indicating that, on average, 2-AG levels neither increased nor decreased significantly within condition.^2^ The magnitude of proportional change in 2-AG in response to alcohol was not significantly correlated with BrAC concentrations, rho = −0.03, *p* = 0.873.

*AEA.* No differences emerged between Alcohol and Placebo conditions for absolute concentrations or proportional change in AEA, all *p*s > 0.632 (or for OEA or PEA; see Supplemental Material). Therefore, in subsequent analyses we focus only on 2-AG.

### Correlation between 2-AG and subjective effects of alcohol

Given the effects of Alcohol were significant only at T + 110 min, we examined the correlation of proportional change in 2-AG at T + 110 min with subjective responses to Alcohol/Placebo intake both pre- and post- the T + 110 min timepoint. This timepoint coincided with the descending limb of BrAC concentrations.

**Fig. 4:**
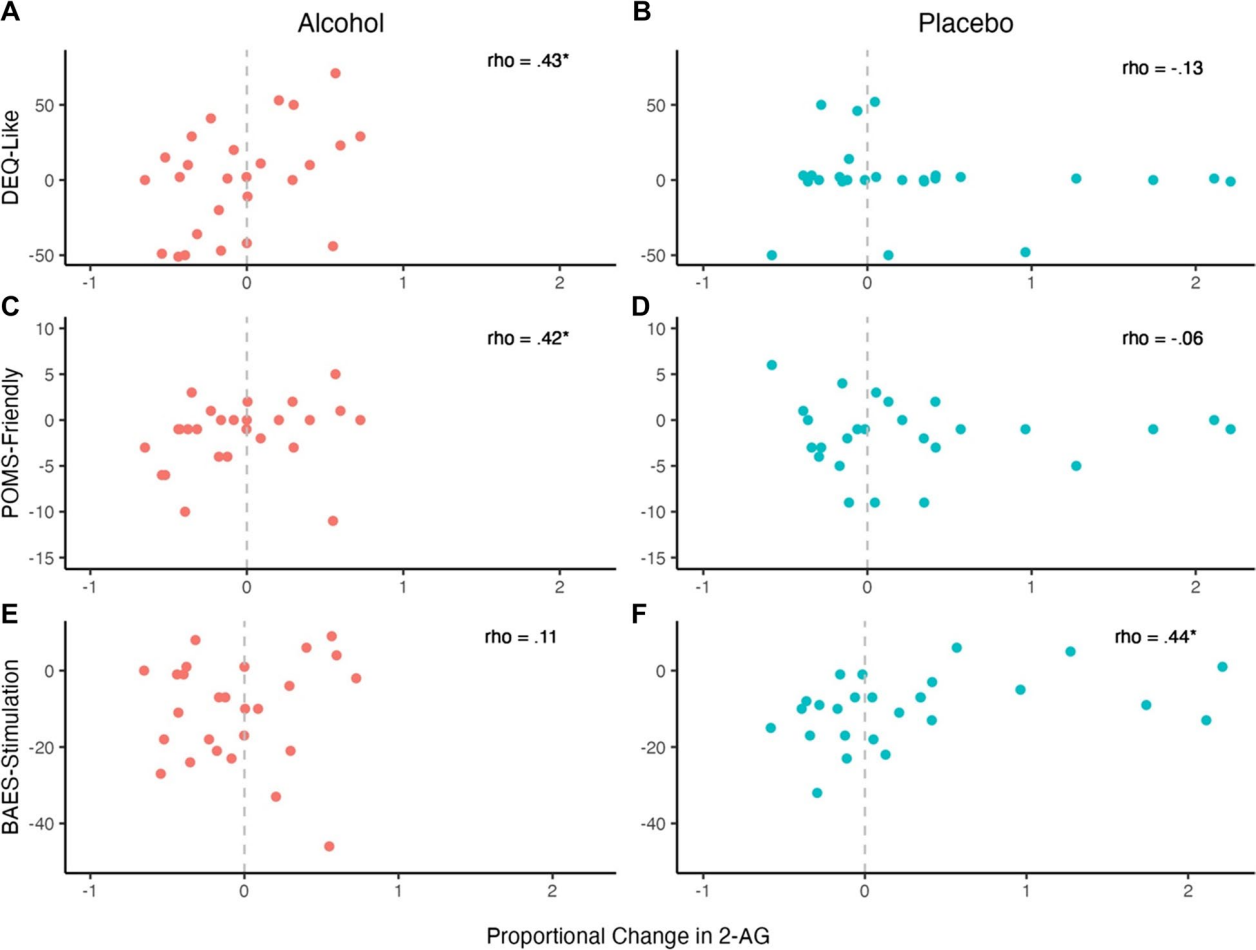
2-AG concentrations were positively correlated with drug liking (DEQ-Like; **A**) and feelings of friendliness (POMS-Friendly; **C**) after the 110 min post-beverage timepoint. In the Placebo condition, 2-AG was positively correlated with feelings of stimulation (BAES-Stimulation) after the 110 min post-beverage timepoint (**F**). Dash line represents no change in 2-AG from baseline. *Spearman correlation *p* <.05

Change in 2-AG concentrations was positively correlated with “liking” the effects of alcohol (DEQ-Like) and feelings of friendliness (POMS-Friendly) at T + 110 min, such that a rise in 2-AG concentrations following alcohol intake was associated with more liking and feelings of friendliness (Fig. 4), rho = 0.43, *p* = 0.027 and rho = 0.42, *p* = 0.033. Stated differently, a *drop* in 2-AG concentrations was associated with less liking and feelings of friendliness. Neither liking nor friendliness were significantly correlated with 2-AG in the Placebo condition, rho = −0.13, *p* = 0.533 and rho = −0.06, *p* = 0.759.

In the Placebo condition, 2-AG concentrations were positively correlated with the stimulation subscale of the BAES (BAES-STIM), such that rise in 2-AG was associated with less of a decrease in feelings of stimulation post-T + 110 min (Fig. 4), rho = 0.44, *p* = 0.025. The correlation was not statistically significant in the Alcohol condition, rho = 0.11, *p* = 0.577.

2-AG concentrations were not statistically significantly correlated with any other post-T + 110 min or pre-T + 110 min subjective effects in the Alcohol or Placebo conditions, rhos < 0.35, *p*s > 0.077.

## Discussion

Our findings from this within-subjects, placebo-controlled laboratory alcohol challenge study, provide novel evidence on the relation between the subjective experience of alcohol intoxication and alcohol-induced changes in peripheral eCB concentrations. Acute alcohol administration was associated with an overall decrease in 2-AG levels compared to placebo at an individual level. We further found that a drop in 2-AG concentrations following alcohol intake was associated with less liking and feelings of friendliness, whereas under placebo conditions, a rise in 2-AG was associated with a smaller decrease in feelings of stimulation (e.g., feeling energized, talkative). Alcohol did not significantly affect AEA concentrations.

Although the primary aim of this study was to investigate the relationship between alcohol-induced changes in eCBs and subjective effects, we began by examining alcohol’s effects on eCB dynamics. This was evaluated through: (1) proportional changes in eCB concentrations compared to baseline levels to assess rising versus falling eCB levels over time; and, (2) differences between alcohol and placebo conditions to isolate alcohol-specific effects. Notably, while alcohol administration led to an overall decrease in 2-AG compared to placebo, the directionality of changes in 2-AG from baseline was not consistent across participants, reflecting individual variability in rising versus falling 2-AG responses to alcohol.

Our findings contribute to a limited literature on alcohol's effects on eCBs in humans (Feuerecker et al. 2012; Joosten et al. 2010; Schrieks et al. 2015; Sloan et al. 2018). Consistent with our results, two prior studies reported significant or trending decreases in 2-AG (Feuerecker et al. 2012; Sloan et al. 2018). However, unlike these studies, we did not observe changes in AEA concentrations. Inconsistent findings in the literature may be attributed to differences in study methodology, including alcohol dose, timing of eCB sampling, and intervening tasks. For example, our study included an MRI scan after alcohol consumption, introducing a minor stress challenge that could have masked alcohol-induced changes in AEA, as stress is known to deplete AEA signaling (Gowatch et al. 2024; Morena et al. 2016). In contrast, other studies incorporated meals and mood inductions into study procedures which could have influenced eCB metabolism and subjective feelings (Joosten et al. 2010; Schrieks et al. 2015). Future studies should experimentally test these procedural differences to understand context-dependent effects of alcohol on eCB dynamics.

The dose of alcohol and timing of eCB sampling also warrant consideration. In the current study, alcohol reliably induced intoxication, confirmed by BrAC and subjective measures, and produced the expected biphasic pattern of stimulation followed by sedation. Peak changes in 2-AG occurred during the descending limb of the BrAC response, typically associated with sedative effects. Previous studies have used lower alcohol doses (Feuerecker et al. 2012; Schrieks et al. 2015) and did not sample eCBs at different points in the biphasic response (Feuerecker et al. 2012; Sloan et al. 2018), limiting comparisons to our findings.

Regarding the study’s primary aim, we found that changes in 2-AG were associated with the subjective effects of alcohol. During the sedative phase of the alcohol response, a drop in 2-AG was associated with less liking and reduced feelings of friendliness, whereas a rise in 2-AG—more akin to the placebo response—was associated with greater liking and friendliness. Under placebo conditions, a rise in 2-AG was linked to smaller drop in feelings of stimulation. Taken together, these results suggest that 2-AG dynamics under normal conditions may support sustained attention, as the observed changes in 2-AG coincide with the MRI scan portion of the session protocol, during which participants are lying down and may need to exert effort to stay focused on the study tasks. In contrast, individuals who continue to mobilize 2-AG in response to alcohol may experience more rewarding subjective feelings, which could contribute to increased motivation to drink.

Interestingly, we did not find a relationship between eCBs and the sedative effects of alcohol or “wanting”. This could indicate that AEA, rather than 2-AG, plays a greater role in mediating these effects. However, as alcohol did not significantly affect AEA levels in this study, further research is needed to clarify its role, particularly in populations at-risk for harmful alcohol use. Notably, correlational studies have linked AEA with harmful alcohol use, further underscoring the need for research on the acute effects of alcohol in at-risk groups (Best et al. 2021; Sipe et al. 2002; Sloan et al. 2018).

Importantly, the variability in 2-AG responses and subjective experiences observed across individuals may reflect a complex interplay of underlying factors. Genetic differences affecting enzymes involved in 2-AG synthesis and degradation (such as DAGL or MAGL, respectively) could influence the magnitude and direction of 2-AG mobilization. Environmental influences, including prior alcohol exposure, stress history, or co-occurring mental health conditions like anxiety, might also shape individual eCB system responsiveness and subjective alcohol effects. Additionally, neurobiological factors such as variability in cannabinoid receptor expression or sensitivity could moderate how 2-AG signaling translates into subjective experience. These inter-individual differences suggest that a “one-size-fits-all” approach to targeting the eCB system in alcohol-related disorders may be insufficient, highlighting the need for personalized therapeutic strategies.

Our findings suggest that atypical 2-AG dynamics may predispose certain individuals to harmful alcohol use. Specifically, heightened hedonic responses to alcohol driven by elevated 2-AG levels may reinforce drinking behavior, increasing the risk of harmful alcohol use, including alcohol use disorders (AUD). Preclinical research supports this hypothesis, showing that dysregulated 2-AG signaling is associated with heightened anxiety and increased alcohol consumption in rodents (Sánchez-Marín et al. 2022a, 2022b). Additionally, post-mortem studies have identified reduced 2-AG levels in individuals with AUD, suggesting long-term alterations in the eCB system due to chronic alcohol use (Sloan et al. 2019). While these findings provide a compelling basis for further investigation, they remain speculative. Longitudinal studies are needed to determine whether alcohol-induced changes in eCBs and their subjective effects predict future patterns of heavy drinking and alcohol use problems.

Our findings, although focused on acute responses in a healthy population, may have important implications for understanding the eCB system as a potential therapeutic target in alcohol use. Specifically, the observed alcohol-induced suppression of 2-AG aligns with growing evidence that alcohol disrupts eCB signaling, which could contribute to dysregulated stress responses and heightened alcohol motivation over time. Emerging preclinical and clinical research suggests that cannabinoid based pharmacological interventions—particularly through administration of phytocannabinoids and inhibition of eCB degradation—may attenuate alcohol intake, reduce relapse-like behavior, and mitigate alcohol-induced harm (Spanagel 2020; Zimmermann et al. 2025). These reports support the view that cannabinoid-based interventions, such as MAGL/FAAH inhibitors, phytocannabinoid administrations, or lifestyle approaches that enhance eCB levels, may hold promise in AUD prevention and treatment strategies. Future research should explore whether alcohol-induced reductions in 2-AG, as seen here, could serve as a biomarker for identifying individuals at risk of problematic drinking or relapse.

Our findings should be interpreted considering the following limitations: First, this study was conducted in healthy social drinkers, limiting generalizability to at-risk populations for harmful alcohol use. Second, eCBs were measured in plasma, which may not accurately reflect central eCB signaling. This is a common limitation in translational research, where animal models assess eCBs directly in the brain, while human studies rely on peripheral measurements (Mayo et al. 2018; Mazurka et al. 2024). Third, while we explored order effects in sensitivity analyses, the study’s small sample size limited the power to detect such effects reliably. The limited statistical power further constrained our capacity to apply corrections for multiple comparisons. Additionally, apart from a negative urine toxicology, we did not assess participants’ self-reported recent or chronic cannabis use, which may influence baseline eCB signaling and anxiety measures, potentially confounding our findings (Gowatch et al. 2024). Future studies should incorporate detailed cannabis use histories to better isolate the specific effects of alcohol and expectancy on the eCB system. While these results should be interpreted with caution, this study addresses a critical gap in understanding the role of the eCB system in alcohol consumption by experimentally examining acute alcohol-induced changes in eCB dynamics and their relationship to alcohol’s rewarding effects.

In summary, this study provides novel evidence linking acute alcohol-induced changes in 2-AG to subjective rewarding effects in humans. These findings suggest that eCB dynamics may contribute to the subjective experience of alcohol intoxication, and therefore, could help explain the eCB system’s role in harmful alcohol use. Future research should explore these mechanisms of acute alcohol intake in at-risk populations and investigate whether eCB dynamics predict patterns of alcohol consumption over time.

## Supplementary information

Below is the link to the electronic supplementary material.

Supplementary file1 (PDF 80 KB)

Supplementary figure 1 (PNG 192 KB)

High Resolution Image (TIF 126 KB)

Supplementary figure 2 (PNG 171 KB)

High Resolution Image (TIF 126 KB)

## Data Availability

Data are available from the corresponding author upon reasonable request.

## Footnotes

1 Normality tests of eCB and subjective feeling parameters indicated that data distributions were mostly non-Gaussian; therefore, we used non-parametric tests for our analyses. Although mixed models were considered, the number of predictor variables relative to our sample size raised concerns about potential overfitting. Nevertheless, we ran mixed models as a sensitivity analysis, and the results remained unchanged.

2 In other words, alcohol did not produce a consistent directional change in 2-AG across participants. However, as noted, when comparing conditions, Alcohol led to a relative reduction in 2-AG concentrations at the individual level, suggesting variability in response direction but an overall reduction when contrasted with Placebo.
