# Supplementary material for: Effects of acute alcohol administration on endocannabinoids and relation to subjective effects
Source: Psychopharmacology (Berl). 2025 Jul 25;243(2):401–11. doi: 10.1007/s00213-025-06843-6 (PMC12904980; doi:10.1007/s00213-025-06843-6)
Supplement: Supplementary file 1 — Supplementary file1 (PDF 80 KB) [file 213_2025_6843_MOESM1_ESM.pdf]

Supplemental Material

Supplemental Results

Change in Sedation and Stimulation Subjective Effects Over Time for Alcohol versus Placebo Conditions

Participants reported greater stimulation on the BAES in the Alcohol condition compared to Placebo at T+30min, $Z = 2.37$, $p = .018$. There were no significant differences at any other time points, Zs < |1.07|, *p*s > .284.

Sedation as reported on the BAES peaked at T+150min in both Alcohol and Placebo conditions, though this comparison was not statistically significant between conditions, $Z = 1.82$, $p = 0.068$, likely reflecting the effects of the MRI scan (see Supplemental Figure 1). Participants reported greater sedative effects on the BAES in the Alcohol condition compared to Placebo at T+15min, $Z = 3.83$, $p < .001$, T+30min, $Z = 3.63$, $p < .001$, and T+180min, $Z = 2.18$, $p = .029$. Conditions did not differ at T=0min, $Z = 1.47$, $p = .141$.

[Insert Supplemental Figure 1]

Change in OEA and PEA Over Time for Alcohol versus Placebo Conditions

No differences emerged between Alcohol and Placebo conditions for absolute concentrations or proportional change in OEA or PEA, all *Z*s < |-1.84|, *p*s > .065 (Supplemental Figure 2).

[Insert Supplemental Figure 2]

Supplemental Figure Legends

Supplemental Figure 1. Feelings of stimulation (BAES-STIM) were greater in the Alcohol condition compared to Placebo 30 min after beverage consumption (left). Feelings of sedation (BAES-SED) peaked 150 min after beverage consumption in both Alcohol and Placebo conditions (right), though this comparison was not statistically significant between conditions. Figure depicts median BAES-Stimulation and BAES-Sedation. Error bars denote standard error of the median. *$p < .05$ Wilcoxon Signed-Rank Test

Supplemental Figure 2. OEA and PEA concentrations did not differ between Alcohol and Placebo conditions at any timepoint. Figure depicts median absolute change in OEA (left) and PEA (right) concentrations relative to baseline levels. Dash line represents baseline. Error bars denote standard error of the median. *$p < .05$ Wilcoxon Signed-Rank Test
