# Supplementary figures and images for: Effects of acute alcohol administration on endocannabinoids and relation to subjective effects
Source: Psychopharmacology (Berl). 2025 Jul 25;243(2):401–11. doi: 10.1007/s00213-025-06843-6 (PMC12904980; doi:10.1007/s00213-025-06843-6)

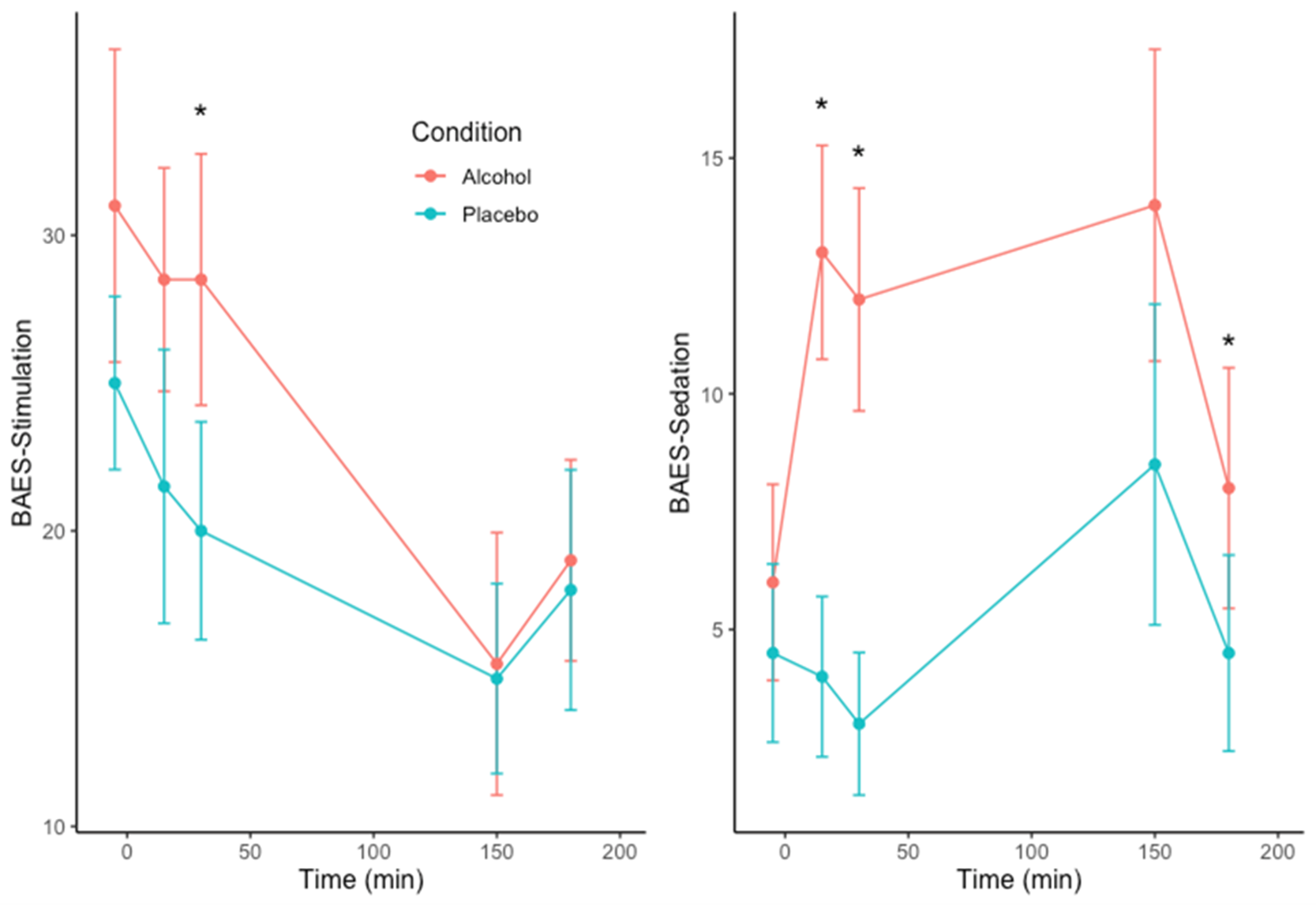

Supplement: Supplementary file 2 — (PNG 192 KB) [file 213_2025_6843_Fig5_ESM.png]

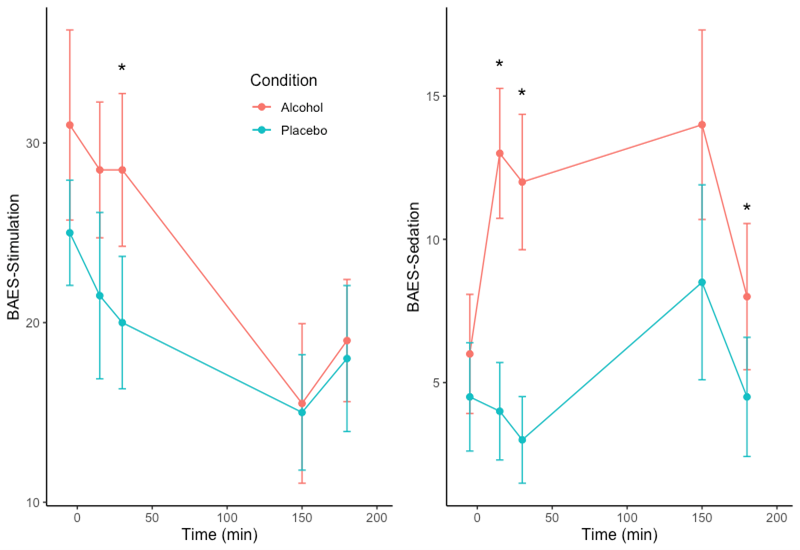

Supplement: Supplementary file 3 — High Resolution Image (TIF 126 KB) [file 213_2025_6843_MOESM2_ESM.tif]

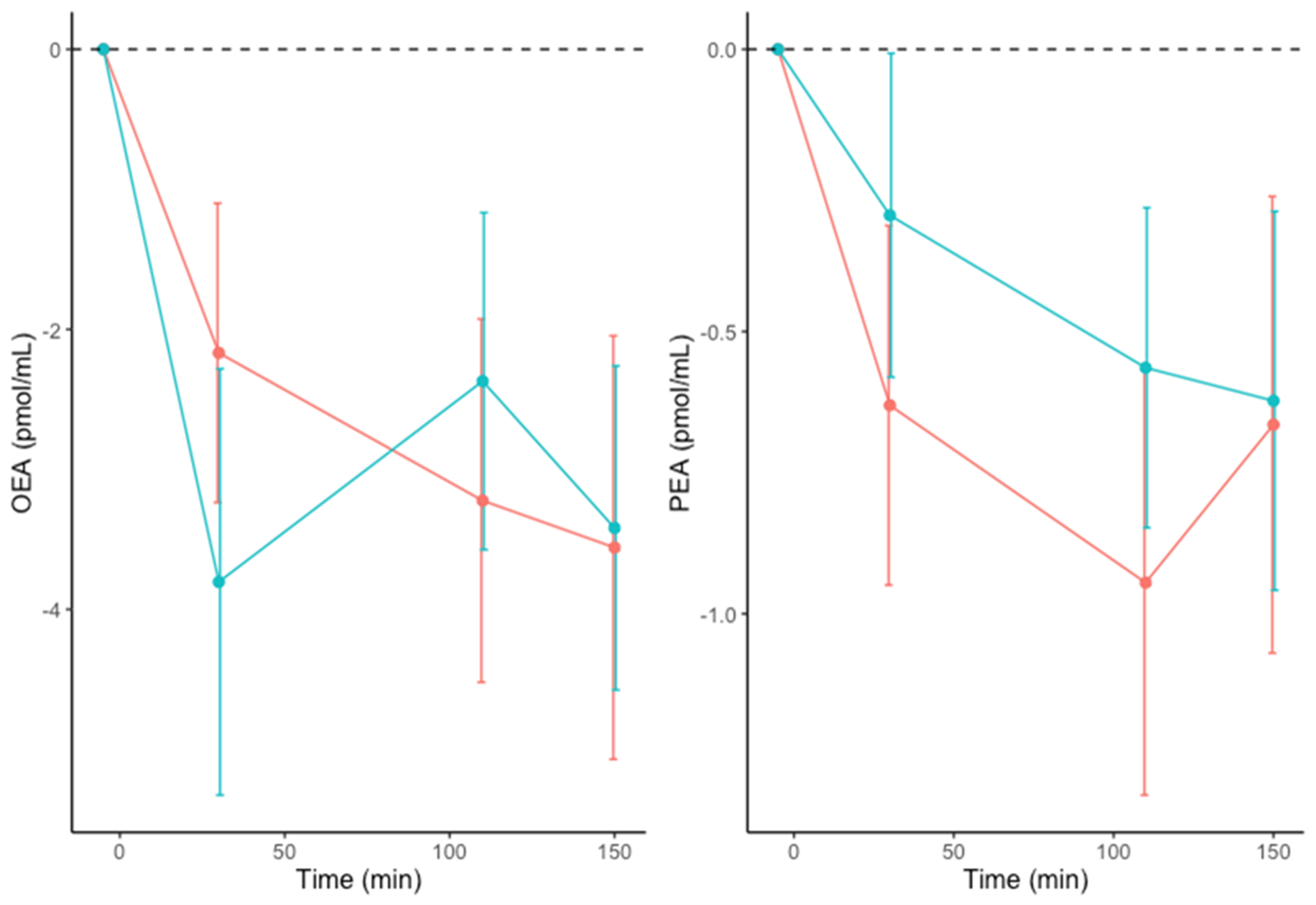

Supplement: Supplementary file 4 — (PNG 171 KB) [file 213_2025_6843_Fig6_ESM.png]

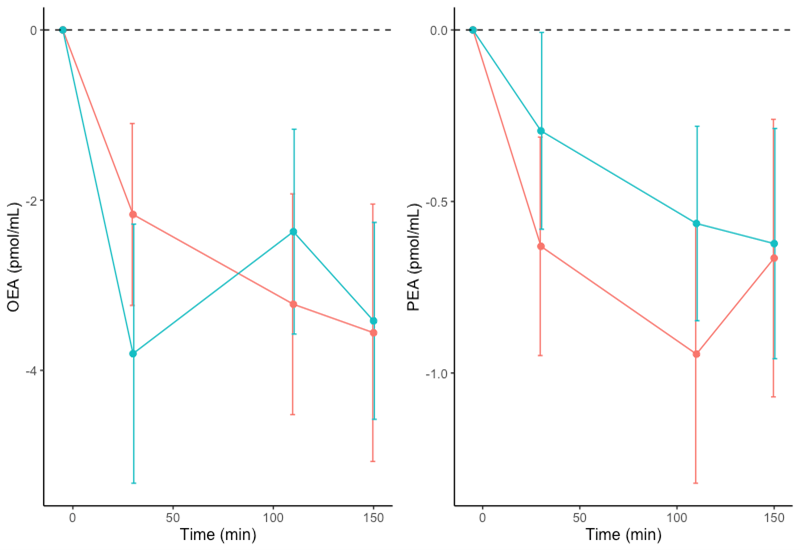

Supplement: Supplementary file 5 — High Resolution Image (TIF 126 KB) [file 213_2025_6843_MOESM3_ESM.tif]
